# Creating an empirically-based model of social arts as a public health resource: Training, typology, and impact

**DOI:** 10.3389/fpubh.2022.985884

**Published:** 2022-10-12

**Authors:** Noa Shefi, Hod Orkibi, Ephrat Huss

**Affiliations:** ^1^Drama and Health Science Lab, and the Emili Sagol Creative Arts Therapies Research Center, Faculty of Social Welfare and Health Sciences, University of Haifa, Haifa, Israel; ^2^Arts in Social Work Masters Specialization, Department of Social Work, Ben-Gurion University, Beer Sheva, Israel

**Keywords:** public health, social arts practice, arts therapies, social work, social change

## Abstract

**Introduction:**

Mounting empirical evidence underscores the health benefits of the arts, as recently reported in a scoping review by the World Health Organization. The creative arts in particular are acknowledged to be a public health resource that can be beneficial for well-being and health. Within this broad context, and as a subfield of participatory arts, the term *social arts* (SA) specifically refers to an art made by socially engaged professionals (e.g., artists, creative arts therapists, social workers, etc.) with non-professionals who determine together the content and the final art product (in theater, visual arts, music, literature, etc.) with the aim to produce meaningful social changes. SA can enhance individual, community, and public health in times of sociopolitical instability and is an active field in Israel. However, SA is still an under-investigated field of study worldwide that is hard to characterize, typify, or evaluate. This paper presents a research protocol designed to examine a tripartite empirically-based model of SA that will cover a wide range of SA training programs, implementations, and impacts. The findings will help refine the definition of SA and inform practitioners, trainers, and researchers, as well as funding bodies and policymakers, on the content and impact of SA projects in Israel and beyond.

**Methods and analysis:**

This 3-stage mixed methods study will be based on the collection of primary qualitative and arts-based data and secondary, complementary, quantitative data. Triangulation and member checking procedures will be conducted to strengthen the trustworthiness of the findings obtained from different stakeholders.

**Discussion:**

Growing interest in the contribution of arts to individual and public health underscores the importance of creating an empirically grounded model for SA. The study was approved by the university ethics committee and is supported by the Israel Science Foundation. All participants will sign an informed consent form and will be guaranteed confidentiality and anonymity. Data collection will be conducted in the next 2 years (2022 to 2024). After data analysis, the findings will be disseminated *via* publications and conferences.

## Introduction

Mounting empirical evidence underscores the health benefits of the arts, as recently reported in a scoping review by the World Health Organization (WHO) (1) and a compilation of over 80 articles on the role of the arts in enhancing a wide range of psychological and physiological health-related outcomes such as socio-emotional skills, mental health, symptoms of trauma, chronic medical conditions, and motor skills (2). The creative arts have been widely acknowledged to be a public health resource that can help promote health and well-being holistically in both individuals and communities, across the lifespan (3–7).

Within this broad context, the social arts (SA) constitute a flourishing subfield of participatory arts that integrates both arts and social practice by crossing disciplinary boundaries. Our preliminary definition of SA is an art made by socially engaged professionals (e.g., artists, creative arts therapists, social workers, etc.) with non-professionals who determine together the content and the final art product (in theater, visual arts, music, literature, etc.) with the aim to produce meaningful social changes. Given the bottom-up approach of the proposed study, this preliminary definition is expected to be refined in light of the findings.

SA can help maintain and enhance individual and collective resilience, empowerment, social inclusion and cohesion and community building in times of sociopolitical instability (8–10). SA is an especially active field in Israel where numerous SA projects have been initiated by artists, social activists, communities, municipalities, agencies, non-governmental organizations (NGOs), and training programs in response to ongoing traumas of war, immigration, marginalization, and intense cultural and religious conflicts. Overall, SA projects fall on a continuum that ranges from enhancing marginalized groups and underserved communities, through humanizing healthcare and institutional settings, to challenging politics. On the **community** end of the spectrum, SA aims to destigmatize marginalized groups and empower their resilience. This can be achieved by giving a symbolic “space for agency” as well as developing critical consciousness (11), similar to the aim of Boal's Theater of the Oppressed, in which a scene of oppression is presented and then replayed with the audience that improvises alternative solutions (12). Traditional arts practices, such as hip-hop, are another way to empower marginalized ethnic groups (13). SA can also help these groups challenge oppression and hegemonic systems by advocating for social critique, community solidarity, civic engagement, and inclusion. Arts can also be used to help negotiate social conflicts in non-violent ways, advocate for citizens' rights and resist injustice (14, 15). SA in **healthcare and institutional** settings (e.g., hospitals, daycare centers, hospices, prisons, and schools) aims to enhance the resilience of healthcare users and providers, and to humanize the cultural climate of institutions (16–18). Finally, on the **political** end of the spectrum, SA utilizes the embodied esthetic elements of the arts to elicit empathy and to change hegemonic attitudes (19). Social art is characterized by the ability to use limited resources creatively that focus the attention of power-holders and engage media attention holders (20, 21). However, this wide spectrum of SA practices–from communities to politics–has not been systematically studied and the multiple aims, practices, scope, and populations of SA project have yet to be characterized and evaluated.

### Characterization of SA training

SA as an emerging interdisciplinary field has been integrated into both arts and social educational practices worldwide (22–24). Increasing numbers of social work curricula include courses in the arts, and in 2008 the first M.A. specialization in the arts in social work was launched at Ben Gurion University of the Negev. Similarly, art schools include SA courses and projects in their training programs; for example, at the Bezalel Academy of Arts and Design, the University of Haifa's School of the Arts, and the Musrara School for Art and Society. Similar trends can be observed in creative arts therapies programs across the country. Each of these programs and institutions is likely to teach SA differently, based on its own orientation. Thus, a theater group using playback (in which audience members tell stories about their own lives and then watch actors bring them to life on the spot) might focus on the esthetic value of outcomes, whereas a social work program using playback might deal with local socio-political issues and can be celebratory and/or critical, by working to debate, reaffirm or undermine sociopolitical norms (25).

The interdisciplinary nature of SA thus challenges the central discourses of both arts and social practice education and creates a new esthetic and social practice paradigm that remains under-investigated to date. For example, the increasing inclusion of SA projects in **creative arts therapies** training programs shifts the paradigm of these courses away from being a “psychology” based on decontextualized biological, dynamic and humanistic theories to include sociopolitical perspectives (26). This changes the setting from a psychotherapeutic space to a social-community setting, and shifts from arts *processes* to include the social presentation of arts *products* (26–33). Further, the inclusion of art challenges the centering of **social work** on “real life” problems, and shifts the paradigm from social sciences to include phenomenological or humanities aims (34). Whereas social practitioners and community workers often utilize photo-voice, projective cards, arts, music, and theater methods extensively, these bottom-up activities are often below the radar of social work research (9, 23, 30, 35).

Finally, including social projects in the **fine arts**, while connecting artists to social contexts, also shifts the paradigm away from the criteria of the esthetic quality and mimetic content of the arts toward social impact. This has been critiqued as delegitimizing fine art as a language in its own right (36–39). In social theater, for example, people who are mainly amateur actors share their stories and views, thus eliminating esthetics and entertainment as the dominant objectives (40–42).

The diversity and multiplicity of SA are thus positive and valuable. The first aim of this study will thus be to systematically compile these different orientations into an integrative and readily accessible tacit and explicit knowledge base that can ultimately promote a better understanding and endorsement of SA training programs by educational decision-makers.

### Evaluating the impact of SA projects

The second aim of this research protocol is to evaluate SA projects. This will make a direct contribution to the creative arts therapies, fine arts, and social work, SA project leaders, participants, and their communities, as well as arts and social practice policymakers who allocate funds for SA projects. As shown above, SA cross disciplinary boundaries and evaluative methods with varying theoretical foundations. Since each SA project has its own specific social agenda, each draws on a theory that pertains to its particular epistemology, location and target population (40, 42). Generally, creative arts therapies research tends to explore the psycho-biological impact of arts on the socially decontextualized individual, rather than the social perspectives of societal impact (28, 29, 43). Similarly, the evaluation of fine arts projects, by definition, does not focus on measuring their social impact, but rather on the innovation and esthetic impact of the end product (36–38). The inclusion of arts in community interventions makes it difficult to evaluate these interventions because their effects are often long-term and the settings are naturalistic and improvised rather than controlled (40, 41, 44).

One way to acquire qualitative and quantitative evidence of the impact of SA is through the evaluation of each project's self-defined outcomes, as has been done in healthcare client-generated outcome assessments (45). Quantitative impact data can also be collected using measures which, according to the literature (46, 47) are appropriate for SA projects, such as collective self-esteem, personal resilience, and personal creative self-efficacy data. This evaluation strategy, together with qualitative data, can capture contextualized as well as more measurable elements of a project's impacts (48).

Another approach to evaluating the impact of arts projects is *arts-based research*, which refers to the systematic use of the artistic processes and outcomes to co-produce knowledge with the study participants through expressive, creative, embodied and phenomenological methods (37). This approach is congruent with the aims of SA, since the arts can be the research subject (as in visual culture), the research method (as in community theater that aims to explore participants' stories), and the research outcome when the artistic product communicates the findings directly back to the community and transforms stances (26). For these reasons, an arts-based procedure along with more formal mixed methods can capture the self-defined impact of SA projects on their participants, as they define them.

### Study aims

This paper presents a research protocol designed to examine a tripartite empirically based model of SA which will cover a wide range of SA training programs, implementations, and impacts using an exploratory bottom-up approach. It has three main aims: (a) characterize SA training programs; (b) map the broadest typology of SA projects possible, including projects initiated and facilitated by artists, social activists, and training programs; and (c) evaluate these projects' impact. These aims correspond with the following research questions:

#### Qualitative and descriptive questions

- How do educators and students participating SA training programs perceive SA in general and the theoretical, methodological, and practice components of SA training in particular?
- What types of SA projects are implemented in Israel? These questions will serve to define the characteristics of SA as a specific field of study and practice.

#### Quantitative question

What is the impact of SA projects on the participants' self-evaluation of their social identity, personal resilience, and confidence in their ability to cope with issues creatively? The response to this question will contribute to the scant evidence on the impact of SA projects on their participants.

#### Mixed methods question

In what ways do the quantitative data contribute to a better understanding of the qualitative data on the impact of SA projects on their participants? This mixed methods analysis will serve to triangulate the data from different sources to strengthen the overall findings.

## Methods and analysis

This 3-stage mixed methods study will combine qualitative and quantitative methods, which is a common practice in the behavioral and social sciences to strengthen the validity of the findings (49, 50). In mixed methods research, the advantages of each method complement one another, providing a better response to the needs of decision-makers. The qualitative components take under consideration the data context and narrative, whereas the quantitative components respond to the need for objective and valid measurements. Specifically, this exploratory study will include an *embedded mixed method design* with a primary qualitative dataset, followed by a secondary quantitative dataset that will play a supportive role in the examination of the impact of SA projects from the preceding phase (51).

### Participants and recruitment

**SA educators (group 1)**, who teach a SA course in creative arts therapies, social work, and fine arts training programs, will be contacted by the research team by email to invite them to participate in the study. If interested, each educator will be asked to (a) provide a curriculum and teaching materials, and (b) take part in an individual online Zoom interview. A minimum of six educators will be recruited.

To recruit **SA students (group 2)**, the research team will send each educator (from group 1 above) an email invitation to participate in the study that the educators will forward to their SA students. Those interested will provide their contact information on the electronic form linked to the email and will be contacted by the research team with a personal invitation to participate in an online group or individual interview *via* Zoom. A minimum of 12 students will be recruited across the creative arts therapies, social work, and fine arts training programs.

**SA project directors (group 3)** will be invited to take part in this study through the authors' existing contacts, a snowball procedure, and internet searches for projects using search terms such as social arts/theater, art/theater for social change, social equality, etc. Those interested will provide their contact information on an electronic form and will be contacted by the research team with an invitation to take part in an online individual Zoom interview. Up to 20 SA project directors will be recruited.

**SA project participants (group 4)** will receive an email from the project manager inviting them to take part in the study. Those interested will provide their contact information on an electronic form and will be contacted by the research team with an invitation to take part in a group interview, online *via* Zoom or face-to-face and will complete a self-report online questionnaire. A minimum of 40 participants across a minimum of 10 SA projects will be recruited.

### Ethical considerations

To address ethical issues, prior to participating in the study, all participants will sign an informed consent form where it is clarified that participation in the study is on a voluntary basis, and anonymity and confidentiality will be guaranteed as well as the right to discontinue participation at any time without penalty or loss of any benefits to which they are otherwise entitled. This study was pre-approved by the University's ethics committee (approval# 549/20).

### Inclusion criteria

The selected training programs must incorporate “hands-on” SA field experience in the program curriculum, rather than consist of theoretical content alone. The SA project should (a) aim to work toward at least one of the following non-exhaustive goals: enhance social resilience, empower individuals of a specific underserved or marginalized community, de-stigmatize and give voice to silenced communities and shift power relations, as well as humanize institutions, help negotiate conflict between groups, act to shift power relations and enhance social justice (15). In addition, the selected SA projects will (b) have operated at least once in the previous 18 months, to minimize participants' memory bias, and will (c) have a visual/audio recording of the final SA product (e.g., exhibition, installation art, performance, concert, etc.).

### Study design and procedure

**Figure 1:**
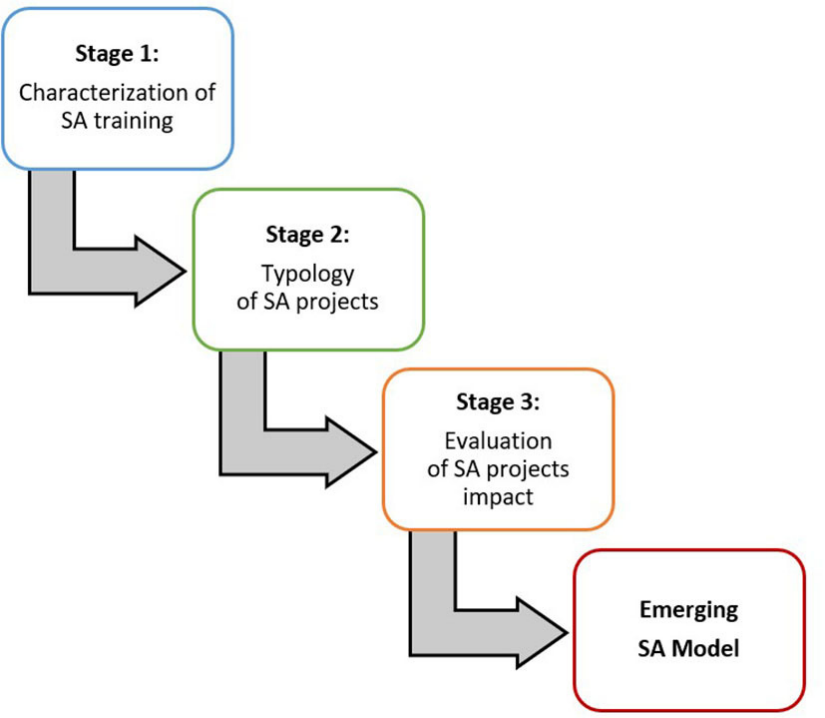
A tripartite empirically based model of SA research.

The research design will have three stages as described below and depicted in Figure 1, consisting of a tripartite empirically based model of SA research. It starts with a characterization of SA training programs, then presents a broad typology of SA projects (including projects initiated and facilitated by artists, social activists, communities, municipalities, agencies, NGOs, and training programs), and culminates in the evaluation of the projects' impact. Each component will ultimately contribute to building a tripartite empirically based model of SA that can inform training courses, implementers, and policymakers. The method summary is shown in Table 1.

**Table 1:** Method summary.

**Research stages**	**Field**	**Method**
Stage 1: Characterizing SA training	Eight cases: 3 fine arts (drama and art) 3 arts therapies (drama and art) 2 social/community work Inclusion criteria: hands-on SA field experience included in the curriculum.	1) Collect and analyze program materials and curricula. 2) Semi-structured interviews with SA educators and students.
Stage 2: Creating a typology of SA projects	Identify at least 20 projects in Israel Inclusion criteria: 1) project on a social theme 2) operated at least once in the last 18 months 3) have a visual/ audio/video recording of the final SA product.	1) Semi-structured interviews with each project director 2) Observation and field notes from ongoing projects 3) Member checking procedure on the accuracy and completeness of the findings.
Stage 3. Evaluation of the impact of SA projects	Select 10 ongoing projects reflecting range of types.	1) Qualitative and arts-based data. 2) Quantitative data Mixed methods analysis.

#### Stage 1: Characterizing SA training

In the first stage of this study (first 12 months) we will analyze and characterize the theoretical, methodological, and practical components of the SA training courses. To this end, we will collect and compare program materials and curricula from six SA courses that meet the above-mentioned inclusion criteria: two in fine arts programs, two in arts therapies programs, and two in social work programs. Next, we will conduct semi-structured interviews with each SA course educator. Then, we will conduct a semi-structured interview with a convenience sample of three to four SA students from the final year of each program, to examine students' overall perceptions of the content and fulfillment of the training course objectives as defined in their SA curricula.

#### Stage 2: Creating a typology of SA projects

The second stage is expected to take up to 18 months and will identify SA projects carried out in Israel over the last 5 years. We will include 10–20 projects, depending on the breadth and depth of each project, to create an empirically grounded typology of SA projects that includes both training and non-training related projects, by drawing on existing contacts with artists, social activists, communities, municipalities, agencies, NGOs, and training programs, as well as through the internet and other documentation.

For each SA project, data from two sources will be collected to strengthen the study's rigor and validity. The first source of data will be semi-structured interviews with each project director. The aim will be to identify and characterize the SA intervention in terms of its initiators, facilitators, participants, and the wider community, its objectives and theoretical orientations, the duration, scope, and arts module used, the assessment of outcomes and funding sources and budgets. The second source of data will be observations and field notes from ongoing projects operating during the time of the study. We will conduct observations of real time meetings, studio/ rehearsal sessions, performances, etc. as a function of each project's phase. To ensure consistency, a single observation will be conducted by the same observer using an observation coding sheet. These observations are expected to provide an in-depth understanding of the projects and enhance the findings' credibility.

A **member checking procedure** will be conducted to minimize confirmation bias and to increase the credibility and validity of our triangulated interpretations and conclusions of this stage (52). To this end, project directors, facilitators, and participants will be invited by email to provide feedback on the accuracy and completeness of our draft of the summary report of the findings. The research team will modify the report accordingly. The empirically grounded typology that will emerge in this stage will represent a bottom-up “map” of the SA field in Israel.

#### Stage 3: Impact evaluation of SA projects

Based on the data gathered in stages 1 and 2, Stage 3 will select at least 10 ongoing SA projects reflecting different types (50% of those identified in Stage 2). The analytic process for this stage will be predominantly inductive, using primary qualitative and arts-based data and secondary, complementary, quantitative data. The collection of both datasets is expected to last for at least 18 months and to provide a diversified and more in-depth understanding of whether and how each SA project impacted all actors involved (53).

### Qualitative data collection

Qualitative data will be collected from multiple contextualized perspectives (54–56), utilizing a participatory arts-based method that was validated in a previous protocol (26). Specifically, for at least 10 ongoing SA projects, we will conduct a participatory 4-h interactive group workshop with about 8–12 participants including the project's initiators, directors, facilitators, participants, and stakeholders (a total of about 80–120 participants). The following procedure will be applied:

1. The group as a whole will observe the final artistic product created in the SA project in a visual/audio/video “stimulated recall” procedure (57) in which the final SA product will serve as a stimulus (e.g., exhibition, concert, performance, etc.).
2. In smaller subgroups, they will be asked to retrospectively reflect and talk about (a) meaningful experiences during the project process and outcomes, both positive and negative; (b) meeting their self-defined objectives; and (c) the project's impact.
3. Then, each subgroup will create a new arts-based product (e.g., visual/ audio/ video) describing the content that emerged in the previous phase. This procedure is expected to expand, deepen, and further elaborate emerging themes in that arts-based work can offer more opportunities for inner exploration and fuller expression and interaction (58). The arts-based product and a list of themes/ issues will be presented to the larger group as a whole.
4. The group will then together define what worked and what did not and for whom and suggest how to improve future SA projects.

These sessions will be audio recorded and transcribed to thematically analyze the participants' verbal reflection on their SA project and arts-based product.

Another **member checking procedure** will invite participants to provide feedback on the accuracy and completeness of the findings (52). In this stage we will email a draft summary of our findings of all the projects identified in Stage 2, in order to assess transferability, which will indicate if the feedback from participants *not* included in Stage 3 supports the confirmability of our interpretations and conclusions (52). The summary will also be sent to at least three SA experts in Europe and the US to further assess their transferability and credibility beyond the Israeli context.

### Quantitative data collection

Complementary quantitative data will be collected along with the primary qualitative data using a web-based survey tool (e.g., Qualtrics) from ongoing SA projects at two different time points that will be determined based on the length of each project and through consultation with each project team.

#### Sample size

To calculate the required sample size for a paired sample *t*-test (comparing two means that are from the same individuals, pretest-posttest) a G^*^power program was used (59). The calculation indicated that for a one-way repeated measure analysis of variance (within-subjects design), a total sample size of at least 40 individuals would be sufficient to detect a medium effect size, defined as 0.06 by Cohen (60). The input parameters for the sample calculation were a medium effect size of $\eta_{\text{p}}^{2}$ = 0.06, alpha = 0.05, power = 0.80, number of groups = 1 (single group design), and number of measurements = 2 (pre-post).

#### Quantitative measures

In addition to the SA projects participants' socio-demographic data (gender, age, ethnicity, religion, and education), specific project-generated outcomes will be defined according to each project's goals, similar to the *Personal Questionnaire*, using a 5-point Likert scale (45). Three additional instruments will be administered for all projects. Collective self-esteem will be measured on the 16-item *Collective Self-Esteem Scale* measuring individuals' collective identity based on their membership in a particular social group (61). The group specification will be modified for each project. Items will be rated on a 5-point Likert scale, with higher scores indicating higher CSE. The Hebrew version has demonstrated good validity and internal consistency reliability with a Cronbach's alpha = 0.70 (62). Personal resilience will be measured on the 6-item *Brief Resilience Scale* (63). We will modify the scale's reference according to each project population. For example: “I tend to bounce back quickly after experiencing discrimination.” The items will be rated on a 5-point Likert scale, with higher scores indicating greater ability to recover from stress (63). This measure has demonstrated good validity and internal consistency reliability with a Cronbach's alpha = 0.70. *Creative self-efficacy* will measure each participant's perceived ability to be creative (64), with a modified creativity reference according to each project's art form (i.e., visual art, drama, music). The six items will be rated on a 5-point Likert scale with higher scores reflecting higher creative self-efficacy. The measure's Hebrew version has demonstrated good validity and internal consistency reliability with a Cronbach's alpha = 0.70 (65).

### Data analysis

The qualitative aim of this study will be to identify themes, commonalities, differences and patterns across all participants and stakeholders (49, 66). After recording and transcribing all the interviews and group sessions, the findings will be thematically analyzed to identify patterns of characteristics of SA as reflected in the participants' experiences (67). One researcher will lead the initial qualitative data analysis, and the two other researchers will oversee the process and check the themes and participants' quotations against the raw interview transcripts. The thematic analysis will follow the six phases outlined by Braun and Clarke (67): preparatory organization, generation of categories or themes, coding data, testing emerging understanding, searching for alternative explanations, and writing up the report.

The analysis will generate a thematic map which will describe the logical relationships between the themes identified by the researchers (68). Through simultaneous data collection and analysis, conducted in an iterative process of moving back and forth between the empirical data and the emerging themes, the data will become progressively more saturated and focused. To strengthen the validity of the findings, a triangulation procedure will be conducted with the qualitative data collected from interviews, curriculum materials, and observation notes (69).

To quantitatively examine the impact of SA projects, the researchers will be assisted by a biostatistician. To examine pre-post changes from two time points for the aggregated scores of all the SA project members, we will conduct a one-way repeated measure analysis of variance (within-subjects design), with 3 levels of hierarchical data consisting of two measures nested within subjects nested within groups.

A mixed methods analysis will integrate the qualitative data and the quantitative data into a whole by comparing and contrasting the results (49). The use of both data methods is expected to provide a diversified and more in-depth understanding of whether and how SA projects impact all participants involved (53).

### Study trustworthiness

The study's trustworthiness will be enhanced not only by triangulation, but also by a member checking procedure that will be conducted to minimize confirmation bias and increase the credibility and validity of our triangulated interpretations and conclusions drawn from Stages 2 and 3 (52). Project directors, facilitators, and participants will be invited to provide feedback on the accuracy and completeness of our summary report of the findings. Memos will be written by the researchers as part of the audit trail, which adds trustworthiness to the analytic process (70).

## Discussion

SA is an emerging interdisciplinary field that extends from empowering underprivileged groups and marginalized communities to challenging politics, and has enormous potential for a positive impact on both individuals and societies (8–10, 53, 71). By weaving together the personal with the social, the arts have the capacity to enhance human capabilities and promote social change, by creating alternative narratives and perspectives (72, 73).

Recently, the multifarious ways in which the arts contribute to health, well-being, individual resilience and enhancing communities (74, 75) has been acknowledged by various professional fields, which have been turning to a wide range of SA projects (30, 76). A report commissioned by the World Health Organization (WHO) in 2019 supports the worldwide development of long-term arts based projects for improving global resilience and well-being (1).

Yet, despite the growing interest in SA, there is almost no empirical evidence on what and how SA is being taught and implemented, or whether and how SA projects are impactful. To date, SA has not been defined in terms of the types of projects initiated, their aims, length, breadth, populations, or their theoretical and methodological characteristics as an integrative whole. Therefore, it is important to create an empirically grounded typology of SA projects, which will take the dominant processes, trends, populations, and themes, as well as variations in theoretical, methodological, and practical orientations into account. The findings will help develop a knowledge base, based on the bottom-up characteristics of varied SA projects, and provide an accessible compilation of SA projects that will be useful to social and arts funding bodies and policymakers, as well as to project initiators and researchers worldwide.

Research in fields related to SA (i.e., creative arts therapies, social work, and fine arts) points to the potential positive impact of the arts on individuals and society (77, 78), but research that measures the specific impact of SA projects is scarce. This study will gather evidence on SA effectiveness by assessing the impact of projects on their initiators, directors, facilitators, and participants, who all require these data to make informed allocations of resources.

Because the emerging field of SA studies has not been sufficiently researched it is important to define the characteristics of SA training programs as a distinct field of study since it has grown out of multiple training directions that include creative arts therapies, social work and the fine arts (visual and theater) (24, 79, 80). The significance of this study lies in characterizing the aims, scope (breadth and depth), and needs in SA training, thus creating an integrative theoretical and methodological model that will be useful to SA teachers, researchers, and practitioners.

Overall, given the scant qualitative and quantitative SA research, the overarching outcome of this study will be to provide a systematic exploration of variations in training, implementation, and the impact of SA projects and generate a tripartite empirically based model of SA. This will ultimately inform practitioners, trainers, and researchers, as well as funding bodies and policymakers, on the content and impact of SA projects, so as to better understand how to maximize project implementations and their impact on society in Israel and beyond.

## Ethics statement

The study involving human participants was reviewed and approved by the Ethics Committee of the Faculty of Social Welfare and Health Sciences, University of Haifa, Israel. Written informed consent for participation was not required for this study in accordance with the national legislation and the institutional requirements.

## Author contributions

NS drafted the text for this protocol, based on the HO and EH conception of the study. All authors have read and approved the final manuscript.

## Funding

This work was supported by the Israel Science Foundation (No. 404/20).

## Conflict of interest

The authors declare that the research was conducted in the absence of any commercial or financial relationships that could be construed as a potential conflict of interest.

## Publisher's note

All claims expressed in this article are solely those of the authors and do not necessarily represent those of their affiliated organizations, or those of the publisher, the editors and the reviewers. Any product that may be evaluated in this article, or claim that may be made by its manufacturer, is not guaranteed or endorsed by the publisher.
